# Association between metabolic dysfunction-associated steatotic liver disease/non-alcoholic fatty liver disease and chronic kidney disease: a systematic review and meta-analysis

**DOI:** 10.1007/s11154-026-10059-0

**Published:** 2026-06-11

**Authors:** André O. Soares, Davi A. C. Loli, Gustavo R. Carvalho, Vinicius Daher Alvares Delfino, Paulo Roberto Bignardi

**Affiliations:** 1 School of Medicine, Pontifícia Universidade Católica do Paraná, Londrina, 86067-000 Paraná Brazil; 2 Departamento de Clínica Médica, Universidade Estadual de Londrina, Hospital Universitário, Londrina, Paraná Brasil

**Keywords:** Chronic kidney disease, Non-alcoholic fatty liver disease, Renal insufficiency, Chronic

## Abstract

Data from current literature have suggested a positive association between Steatotic Liver Disease (SLD) and Chronic Kidney Disease (CKD). In 2023, a consensus of experts suggested that the term metabolic associated steatotic liver disease (MASLD) be used instead of non-alcoholic fatty liver disease (NAFLD), a term that had long been used to indicate the situation of hepatic steatosis not related to alcohol consumption or secondary to another etiology. The majority of hepatologists accepted this suggestion. To critically analyze this described association and compare the impact of change in criteria, we performed a systematic review followed by meta-analysis of incidence studies that evaluated the occurrence of new cases of CKD in patients diagnosed with NAFLD and MAFLD/MASLD. We searched the PubMed, LILACS, SciELO, and Cochrane Library databases for longitudinal studies published through June 2025 that included groups with NAFLD, MAFLD, and/or MASLD, as well as a control group, in the adult population without CKD at baseline. Among 2,099 studies found, twenty met the inclusion criteria. Compared to the patient’s control group, the risk ratio (RR) for incidence of CKD in the group with MAFLD, MASLD, and NAFLD was 1.29 (1.18–1.40), 1.16 (1.12–1.19) and 1.25 (1.14–1.36), respectively. In a new analysis grouping MASLD with MAFLD to assess the impact of the terminology shift, the results remained similar. This review demonstrates that SLD is associated with the risk of developing CKD, regardless of classification. However, this connection needs further elucidation of its mechanisms to comprehend it better and prevent its impact.

**Supplementary Information:**

The online version contains supplementary material available at https://doi.org/10.1007/s11154-026-10059-0.

## Introduction

Nonalcoholic Fatty Liver Disease (NAFLD) has been replaced by Metabolic-Dysfunction-Associated Fatty Liver Disease (MAFLD), and more recently, by Metabolic Dysfunction-Associated Steatotic Liver Disease (MASLD). The last two denominations were introduced to emphasize the role of metabolic disfunction in the pathogenesis of the disorder [1–3]. Because there is an almost complete overlap of patients with MAFLD and MASLD, and since MASLD is the term recommended by various expert societies dealing with the subject, it will be used from now on, unless otherwise specified [4, 5].

Tracking the obesity and diabetes epidemics, the prevalence of MASLD has been increasing progressively in recent years and currently affects about 1/3 of the world’s adult population, being the leading cause of cirrhosis and hepatocellular carcinoma in developed Western countries [3, 6]. Its presence also is associated with increased rates of colorectal cancer [7]. An important warning that the burden of this condition will keep increasing significantly is present in a recent report from Obesity Medical Association (OMA) that estimated that 30–50% of obese children and adolescents have MASLD [6, 8].

MASLD is now considered a multisystemic disease as it is also associated with damage/malfunction in several organs outside the digestive tract, mainly the cardiovascular system and the kidneys [3, 9, 10]. Indeed, recently, MASLD has been suggested to be an independent risk factor for chronic kidney disease (CKD) [2, 11, 12].

Regarding the association with CKD, old studies may have included “lean NAFLD” or misclassified alcohol-related disease, introducing heterogeneity. Similarly, CKD definitions have now become standardized to include abnormal albuminuria in addition to reduced estimated glomerular filtration rate [13]. A new large meta-analysis would reframe the evidence under modern definitions.

Given these considerations, we decided to conduct a systematic review followed by a meta-analysis on a possible association between the presence of NAFLD/MASLD and the incidence of CKD.

## Methods

This systematic review was conducted according to the Preferred Reporting Items for Systematic Reviews and Meta-Analyses (PRISMA) guidelines [14]. It is registered in the international database for prospectively registered systematic reviews (PROSPERO) (ID: CRD42023410188).

### Research strategy

This study was conducted through a search in PubMed, LILACS, SciELO, and the Cochrane Library databases, covering studies published up to June 27th, 2025, in English, Portuguese, and Spanish. Clinical trial registries, including ClinicalTrials.gov, were also explored. The search terms used were: (non-alcoholic fatty liver disease OR Nonalcoholic Fatty Liver OR NAFLD OR non-alcoholic steatohepatitis OR MASLD OR metabolic dysfunction-associated steatotic liver disease) AND (Renal insufficiency, chronic OR chronic kidney disease OR Kidney diseases OR Kidney failure OR estimated glomerular filtration rate).

### Study selection

Two independent authors screened the studies. If there was no agreement, conflicts were resolved through discussions with a third author. After removing duplicates, the articles were reviewed solely by their titles and abstracts to exclude those that did not meet the inclusion criteria, followed by a thorough review of each remaining article. The Rayyan online platform was used for the selection process.

### Exposure definitions

To avoid conceptual overlap, this review adopted explicit operational definitions for each category of steatotic liver disease. NAFLD was defined as the presence of hepatic steatosis in the absence of other known causes of liver disease, reflecting its traditional exclusion-based framework. MAFLD was defined according to a positive diagnostic approach, requiring evidence of hepatic steatosis in the presence of metabolic dysfunction, without the need to exclude other liver disease etiologies. MASLD was considered within the newer steatotic liver disease (SLD) nomenclature and follows a framework conceptually similar to NAFLD, in which alternative causes of liver disease are excluded, representing an updated terminology within this classification system.

In this review, NAFLD, MAFLD, and MASLD were recorded strictly according to the terminology explicitly used in the original studies; no attempts were made to reclassify study populations based on reported metabolic or clinical criteria. Chronic kidney disease (CKD) was considered either when explicitly reported by the authors or when defined according to Kidney Disease: Improving Global Outcomes (KDIGO) criteria, namely an estimated glomerular filtration rate (eGFR) < 60 mL/min/1.73 m² and/or a urine albumin-to-creatinine ratio > 30 mg/g, persisting for more than three months [13].

### Inclusion criteria

Studies were considered eligible if they met the following criteria: (1) included adult participants without chronic kidney disease (CKD) at baseline; (2) evaluated an exposure group defined by NAFLD, MAFLD, or MASLD; (3) included a comparator group without steatotic liver disease; (4) reported incident CKD as an outcome; and (5) had a longitudinal design. No restrictions were placed on sample size.

Exposure definitions were accepted as reported by the original studies, provided that hepatic steatosis was identified by imaging or histology. Histological evidence was defined as the presence of steatosis, either alone or in association with inflammation and/or hepatocellular ballooning [15]. Imaging-based diagnosis was considered when typical ultrasonographic features were present, such as hepatorenal echo contrast, liver brightness, or vascular blurring [16].

The inclusion of studies addressing NAFLD and MASLD required the exclusion of alcoholic liver disease, defined as daily alcohol consumption ≥ 30 g for men and ≥ 20 g for women, as well as the exclusion of other secondary causes of liver disease [15, 17].

For studies addressing MAFLD, the presence of metabolic dysfunction was required in addition to steatosis, including: (1) overweight or obesity, defined as body mass index ≥ 25 kg/m² (≥ 23 kg/m² in Asian populations) and/or waist circumference ≥ 94 cm in men and ≥ 80 cm in women; ≥ 90 cm in men and ≥ 80 cm in women for South Asian and Chinese populations; and ≥ 85 cm in men and ≥ 90 cm in women for Japanese populations; (2) type 2 diabetes mellitus, defined as HbA1c ≥ 6.5%, fasting plasma glucose ≥ 126 mg/dL, 2-hour plasma glucose during oral glucose tolerance test ≥ 200 mg/dL, or ongoing treatment for type 2 diabetes; or (3) evidence of metabolic dysregulation in lean individuals, such as prediabetes (HbA1c 5.7–6.4%, fasting plasma glucose 100–125 mg/dL, or 2-hour plasma glucose during oral glucose tolerance test 140–199 mg/dL), dyslipidemia (plasma triglycerides ≥ 150 mg/dL, high-density lipoprotein cholesterol ≤ 39 mg/dL in men or ≤ 50 mg/dL in women, or lipid-lowering therapy), or hypertension (blood pressure ≥ 130/85 mmHg or use of antihypertensive medication) [17]. Studies using non-standard or unspecified diagnostic methods (e.g., fatty liver index [FLI] or ICD-10 codes alone) were excluded.

Incident CKD was defined either as reported by the study authors or, when not explicitly stated, according to Kidney Disease: Improving Global Outcomes (KDIGO) criteria (eGFR < 60 mL/min/1.73 m² and/or albumin-to-creatinine ratio > 30 mg/g, persisting for more than three months) [13].

### Data extraction

The data extraction was conducted by three independent authors using a form specifically designed for this study. When discrepancies were identified, they were resolved through discussion with a fourth author. The data recorded included authors’ names, year of publication, study design, country, sample size, studied population and demographic data (including other clinical diagnoses), baseline characteristics of these populations, recruitment and retention rates, and risk of CKD, providing odds ratio (OR), risk ratio (RR), or hazard ratio (HR) with 95% confidence intervals (CI). The effect measures extracted were then adjusted for a greater number of confounding variables.

### Results evaluation

First, the analysis focused on the risk of developing CKD in adults with NAFLD, MAFLD, and MASLD. Additionally, it aimed to assess the effects of NAFLD versus MAFLD plus MASLD on the same outcome.

### Studies’ quality assessment

The authors independently assessed the quality of each study. The quality of the included studies was assessed using the Newcastle-Ottawa quality scale [18]. Considering studies with a score of 7 or higher as high-quality, by analyzing their participant selection, comparability, and outcomes, which could result in a maximum score of 9. 

### Data analysis and risk of bias

Effect estimates with the most significant degree of adjustment for potential confounding factors were extracted. Studies included in the meta-analysis reported OR, RR, or HR. HR was compared to RR, while in studies that provided OR, a corrected RR was calculated [19–21]. Pooled RR and 95% CI were estimated using a random effects model (DerSimonian-Laird model with standard-error adjustment using Sidik-Jonkman), based on heterogeneity. Cochran’s Q test was employed to evaluate statistical significance, and the I² statistic was used to assess heterogeneity among studies, with an I² value of 50% or greater indicating substantial heterogeneity. A leave-one-out sensitivity analysis was performed to evaluate the impact of each study. The Egger test and funnel plot were utilized to examine publication bias. All analyses were conducted with Stata/SE v.19.5 (Statacorp LP, USA).

## Results

### Study selection and characteristics

**Fig. 1:**
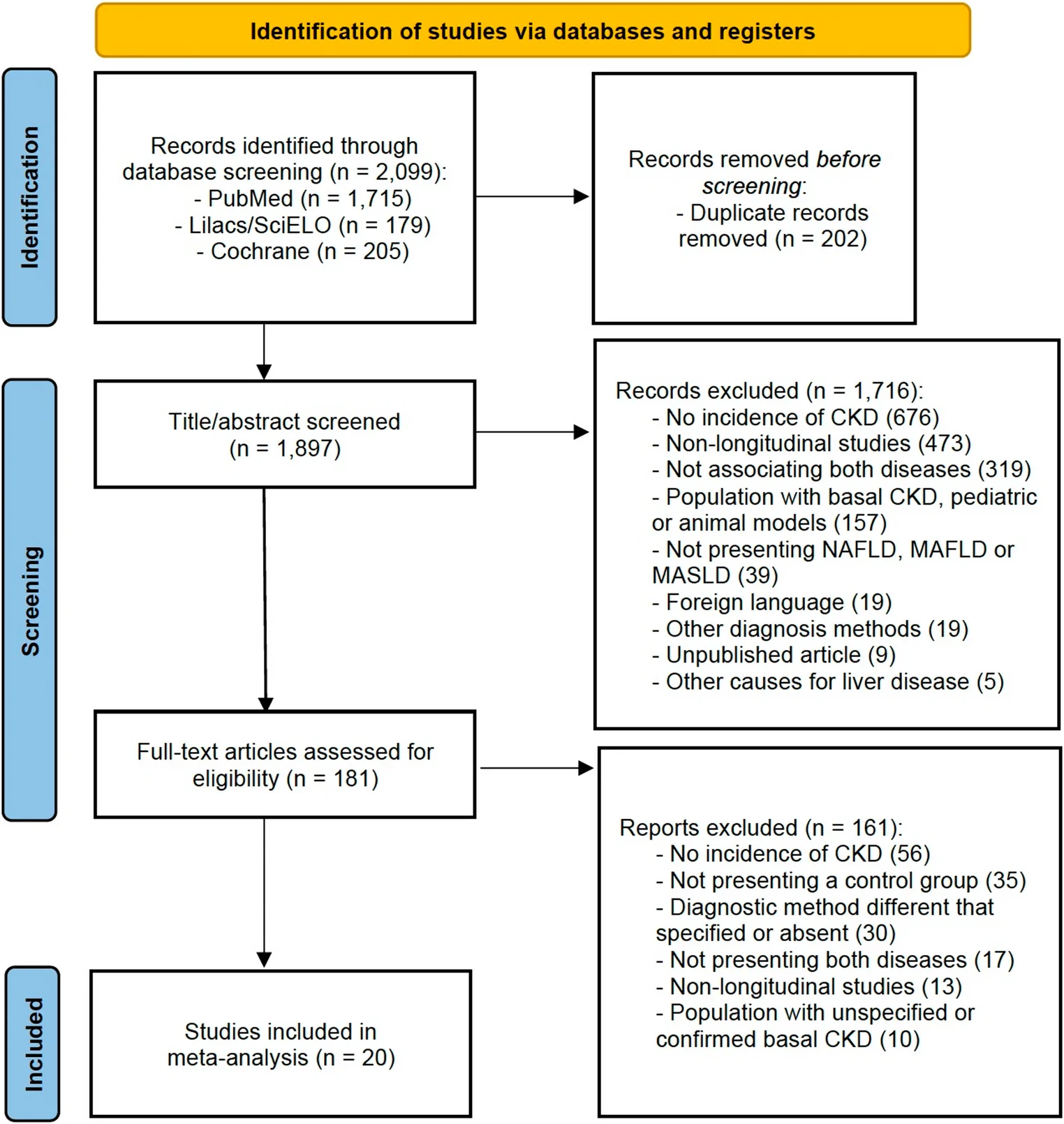
Flow chart of the literature review process

The initial search identified 2,099 studies, with 202 of them being excluded for being duplicates. Studies that did not present NAFLD, MAFLD and/or MASLD, the development of CKD, or did not associate them; had other liver diseases than NAFLD, MAFLD or MASLD; showed only prevalent CKD; non-longitudinal studies, narrative reviews or studies that presented in a foreign language were excluded during the screening. The full-text analysis of the remaining 192 articles excluded studies that, besides previous reasons for exclusion, did not present a control group without NAFLD, MAFLD and MASLD; used alternative diagnostic methods or did not specified them; were composed of non-longitudinal studies; and did not specify the incident CKD population (with or without NAFLD, MAFLD and MASLD), resulting in twenty studies that fulfilled the inclusion criteria (Fig. 1) [22–41]. The basic characteristics of the studies included are shown in Table 1.

**Table 1:** Characteristics of studies selected

Author (year)	Country	Design	Follow-up	Journal	Population	Sample	Patients with NAFLD/ MAFLD/ MASLD	Patients without NAFLD/ MAFLD/ MASLD	Diagnostic method for CKD	Outcomes	Diagnostic method for NAFLD/ MAFLD/ MASLD	Covariates adjustment
Chang et al., 2008	South Korea	Prospective cohorts	3.21 years	Elsevier	Male patients, nonhypertensive and nondiabetic, aged 30 to 59 years	8,329	2,516 with **NAFLD**	5,813 without **NAFLD**	Estimated glomerular filtration rate < 60 mL/min per 1.73 m2 or positive proteinuria	Incidence of CKD	Abdominal ultrasonography	Age, baseline GFR, body mass index (BMI), fasting serum glucose, systolic blood pressure, triglyceride (TG), high-density lipoprotein cholesterol (HDL-C), insulin resistance (HOMA-IR), high-sensitivity C-reactive protein (hsCRP), smoking, alcohol consumption, incident diabetes, and incident hypertension
Gao et al., 2024	China	Prospective cohorts	12.9 years	Journal of the American Heart Association	Participants aged between 18 and 98	79 540	24,083 with **MAFLD** 19,062 with **MASLD**	55,367 without **MAFLD** 52,285 without **MASLD**	Estimated glomerular filtration rate < 60 mL/min per 1.73 m2 or positive proteinuria	Incidence of CKD	Abdominal ultrasonography	Age, sex, smoking habits, drinking consumption, exercise, education, eGFR, uric acid, BMI, hs-CRP, diabetes, hypertension, and dyslipidemia.
Hashimoto et al., 2022	Japan	Retrospective cohorts	4.6 years	Journal of Diabetes Investigation	Participants of a medical health checkup program, after excluding those with CKD at baseline examination.	16,938	3,318 with **MAFLD** 409 with **NAFLD** (FLD without MD)	13,620	Estimated glomerular filtration rate < 60 mL/min per 1.73 m2 or positive proteinuria	Incidence of CKD	Abdominal ultrasonography	Sex, age, alcohol consumption (logarithm-transformed), smoking status, exercise habits, and serum creatinine levels.
Heo et al., 2024	South Korea	Retrospective cohorts	6.1 years	HepatoBiliary Surgery and Nutrition	Adults aged 18 years and older, all with normal kidney function at baseline.	214,145	44,254 with **NAFLD** and **MASLD**	151,759 **No-SLD**	Estimated glomerular filtration rate < 60 mL/min per 1.73 m2 or positive proteinuria	Incidence of CKD	Abdominal ultrasonography	Age, sex, education level, smoking history, regular exercise (≥ 3x/week), daily alcohol intake, prior history of coronary artery disease, use of any antihypertensive medications, and baseline eGFR levels.
Kwon et al., 2023	Republic of Korea	Retrospective cohorts	5.3 years	Nature	Subjects who had at least two serial health examinations and underwent abdominal ultrasonography at the first health examination were identified.	21,713	5,267 with **MAFLD** and **NAFLD** (both FLD)	14,525	Estimated glomerular filtration rate < 60 mL/min per 1.73 m2 or positive proteinuria	Incidence of CKD	Abdominal ultrasonography	Age, sex, estimated glomerular filtration rate, smoking status, physical activity, prediabetes, diabetes, hypertension, cardiovascular disease, NAFLD fibrosis score, and body mass index.
Liang et al., 2022	China	Retrospective cohorts	4.6-Year	Oxford	Subjects aged 55 to 70 years, without CKD at baseline, submitted to a follow-up period.	6,873	2429 with **MAFLD** (with metabolic dysfunction only) 2452 with **NAFLD**	3,311	Estimated glomerular filtration rate < 60 mL/min per 1.73 m2 or positive proteinuria	Incidence of CKD	Abdominal ultrasonography	Age, sex, educational background, smoking status, and leisure-time exercise.
Mikolasevic et al., 2022	Croatia	Prospective cohorts	7,6 years	Elsevier	Middle-aged outpatients with type 2 diabetes (T2DM) without a prior history of myocardial infarction (AMI), stroke (CVI), or chronic kidney disease (CKD)	238	171 with **NAFLD**	67	Estimated glomerular filtration rate < 60 mL/min per 1.73 m2 or positive proteinuria	Incidence of CKD	Abdominal ultrasonography and FibroScan	,Age, sex, body mass index (BMI), hypertension, dyslipidemia, and diabetes-related variables .
Mori et al., 2024	Japan	Prospective cohorts	10 years	The Japan Society of Hepatology	Individuals who received annual health examinations without CKD	11,312	3,396 with **MASLD**	7,916 without **MASLD**	Estimated glomerular filtration rate < 60 mL/min per 1.73 m2 or positive proteinuria	Incidence of CKD	Abdominal ultrasonography	Age, sex, eGFR, current smoking habit, diabetes mellitus, hypertension, dyslipidemia, and Fibrosis-4 index (FIB-4).
Önnerhag et al., 2019	Sweden	Retrospective cohorts	19.5 years	Elsevier	Male (mean age 46 years) and female (mean age 49 years)	1,875	120 with **NAFLD**	1,755 without **NAFLD**	Estimated glomerular filtration rate < 60 mL/min per 1.73 m2 or positive proteinuria	Incidence of CKD	Biopsy proven	Age, sex, type 2 diabetes mellitus, hypertension, and fibrosis stage in analyses involving CKD incidence and mortality.
Saito et al., 2021	Japan	Retrospective cohorts	6 years	Nature	Adult patients with type 2 diabetes mellitus and free of CKD at baseline.	584	96 with **NAFLD**	397 without **NAFLD**	Estimated glomerular filtration rate < 60 mL/min per 1.73 m2 or positive proteinuria	Incidence of CKD	Abdominal ultrasonography	Age, sex, BMI, baseline HbA1c, baseline eGFR, smoking and drinking status (current or past), comorbidities: hypertension, dyslipidemia, anti-diabetic medications and anti-hypertensive medications (e.g., ACEi/ARB).
Seo et al., 2022	Korea	Prospective cohorts	Follow-up of 8.3 ± 3.6 years	Diabetes and Metabolism Journal	Patients with T2DM, preserved renal function	3,188	1729 with **NAFLD**	1,459 without **NAFLD**	Estimated glomerular filtration rate < 60 mL/min per 1.73 m2 or positive proteinuria	Incidence of CKD	Abdominal ultrasonography	Age, sex, body mass index (BMI), duration of diabetes, systolic blood pressure (SBP), baseline HbA1c level, total cholesterol level, estimated glomerular filtration rate (eGFR), use of sulfonylurea, insulin, statins, ACE inhibitors, ARBs, high-sensitivity C-reactive protein (hs-CRP), and insulin sensitivity assessed by KITT values.
Sinn et al., 2017	South Korea.	Retrospective cohorts	4.5 years	Elsevier	Adult men and women without CKD at baseline	41,430	14,223 with **NAFLD**	27,207 without **NAFLD**	Estimated glomerular filtration rate < 60 mL/min per 1.73 m2 or positive proteinuria	Incidence of CKD	Abdominal ultrasonography	Age, sex, year of visit, smoking status, alcohol consumption, BMI, systolic blood pressure, hemoglobin A1c, LDL cholesterol, use of antihypertensive medications, use of antidiabetic medications, and use of lipid-lowering medications.
Tanaka et al., 2022	Japan	Prospective cohorts	10 years	Nephrol Dial Transplant	Individuals with abdominal ultrasonography and without CKD	13,159	4,253 with **MAFLD**	8,906 Without **MAFLD**	Estimated glomerular filtration rate < 60 mL/min per 1.73 m2 or positive proteinuria	Incidence of CKD	Abdominal ultrasonography	Age, sex, eGFR, current smoking habits, ischemic heart disease, diabetes mellitus, overweight/obesity, hypertension, and dyslipidemia.
Targher et al., 2008	Italy	Prospective cohorts	6.5 years	Journal of the American Society Nephrology	Adults with type 2 diabetes, normal or near-normal kidney function, and no overt proteinuria	1760	1289 with **NAFLD**	471 without **NAFLD**	Estimated glomerular filtration rate < 60 mL/min per 1.73 m2 or positive proteinuria	Incidence of CKD	Abdominal ultrasonography	Gender, age, BMI, waist circumference, BP, smoking, diabetes duration, HbA1c, lipids, baseline eGFR, microalbuminuria, and use of medications (hypoglycemic, lipid-lowering, antihypertensive, or antiplatelet drugs).
Targher et al., 2014	Italy	Retrospective cohorts	5.2 years	Diabetes Care	Type 1 diabetic adults with preserved kidney function and with no macroalbuminuria at baseline	261	131 with **NAFLD**	130 without **NAFLD**	Estimated glomerular filtration rate < 60 mL/min per 1.73 m2 or positive proteinuria	Incidence of CKD	Abdominal ultrasonography	Age, sex, diabetes duration, A1C, hypertension, baseline eGFR, and exclusion of microalbuminuria.
Wei et al., 2023 (1)	China	Retrospective cohorts	10 years	Elsevier	Participants diagnosed type 2 diabetes	3,627	2,234 with **MAFLD**	1,393 without **MAFLD**	Estimated glomerular filtration rate < 60 mL/min per 1.73 m2 or positive proteinuria	Incidence of CKD	Abdominal ultrasonography.	Age, gender, hypertension, dyslipidemia, overweight/obesity, baseline eGFR levels, AST, ALT, and LDL cholesterol.
Wei et al., 2023 (2)	China	Retrospective cohorts	10 years	JMIR Public Health Surveil.	Participants who had received at least 3 health examinations and had an abdominal ultrasonography performed from 2008 to 2015	41,246	11,860 with **MAFLD**	29,386 without **MAFLD**	Estimated glomerular filtration rate < 60 mL/min per 1.73 m2 or positive proteinuria	Incidence of CKD	Abdominal ultrasonography	Age, gender, hypertension, dyslipidemia, overweight or obesity, type 2 diabetes, LDL-C, AST, ALT, and creatinine.
Wilechansky et al., 2019	USA	Prospective cohorts	12.5 years	HHS Public Access	Individuals who underwent CT scans and had interpretable liver fat data.	987	189 with **NAFLD**	798 without **NAFLD**	Estimated glomerular filtration rate < 60 mL/min per 1.73 m2 or positive proteinuria	Incidence of CKD	Multidetector computed tomography	Age, sex, smoking status, alcohol consumption, systolic/diastolic blood pressure, use of antihypertensive medications, HDL cholesterol, total cholesterol, diabetes, regular aspirin use, and BMI.
Zhang et al., 2023 (TCLSIH)	China	Prospective cohorts	97,682 person-years	Elsevier	General population in Tianjin, China, who met specific inclusion and exclusion criteria (e.g., no pre-existing CKD, cancer, or cardiovascular diseases).	25,974	7583 with **MAFLD** 7095 with **NAFLD**	18,391 without **MAFLD** 18,879 without **NAFLD**	Estimated glomerular filtration rate < 60 mL/min per 1.73 m2 or positive proteinuria	Incidence of CKD	Abdominal ultrasonography	Age, sex, education level, employment status, household income, energy intake, baseline eGFR, other kidney diseases, and family history of hypertension, cardiovascular diseases, and diabetes.
Zuo et al., 2021	China	Prospective cohorts	4.4 years	Oxford	Participants aged 40 years or older and free of CKD at baseline.	4,042	1,187 with **NAFLD**	2,855 without **NAFLD**	Estimated glomerular filtration rate < 60 mL/min per 1.73 m2 or positive proteinuria	Incidence of CKD	Abdominal ultrasonography	Age, sex, current smoking and drinking status, physical activity, BMI, triglycerides (log-transformed), systolic blood pressure, HbA1c, white blood cell count, UACR (log-transformed), eGFR, use of antidiabetic, antihypertensive, and lipid-lowering medications, as well as changes in diabetes, hypertension, and obesity status during follow-up.

### Association between NAFLD and incident CKD

**Fig. 2:**
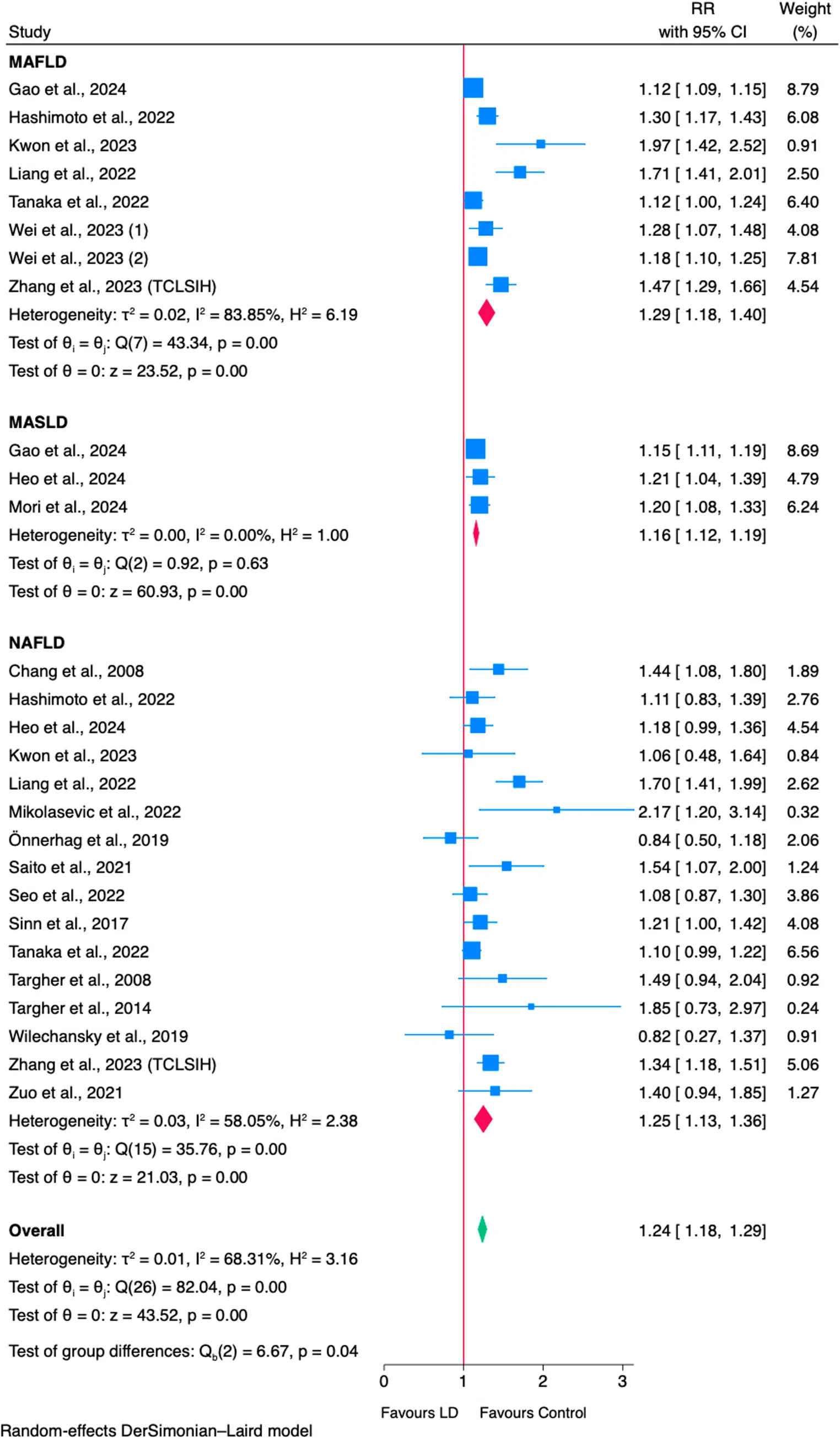
Forest plot for association between NAFLD with CKD [22– [36, 38]; MAFLD with CKD [26, 27, 31, 34], 37– [40]; and MASLD with CKD [22, 37, 41]. CKD: Chronic Kidney Disease; NAFLD: Non-alcoholic Fatty Liver Disease; MAFLD: Metabolic Dysfunction-Associated Fatty Liver Disease; MASLD: Metabolic Dysfunction-Associated Steatotic Liver Disease

The data were extracted from sixteen studies [22–36, 38]. The pooled RR of CKD in the NAFLD group versus the non-NAFLD group was 1.25 (95% CI 1.13–1.36), with statistical significance (*p* < 0.05). Therefore, NAFLD patients were shown to have an increased risk of developing CKD when compared to non-NAFLD patients, as shown in Fig. 2.

### Association between MAFLD and incident CKD

The data were extracted from eight studies [26, 27, 31, 34, 37–40]. The pooled RR of CKD in the group of MAFLD patients versus the non-MAFLD group was 1.29 (95% CI 1.18–1.40), with statistical significance (*p* < 0.05) and heterogeneity (I²=83.85%). In this case, MAFLD patients demonstrated an increased risk of developing CKD when compared to those without a MAFLD diagnosis (Fig. 2).

### Association between MASLD and incident CKD

The data were extracted from three studies [22, 37, 41]. The pooled RR of CKD in the MASLD group versus the non-MASLD group was 1.16 (95% CI 1.12–1.19), indicating that its presence was a predictor for CKD (*p* < 0.05). The analysis showed low heterogeneity (I² = 0.00%). Thus, patients with MASLD demonstrated a higher risk of developing CKD when compared to those without MASLD, as shown in Fig. 2.

### Comparing the associations between MASLD/MAFLD and NAFLD with incident CKD

**Fig. 3:**
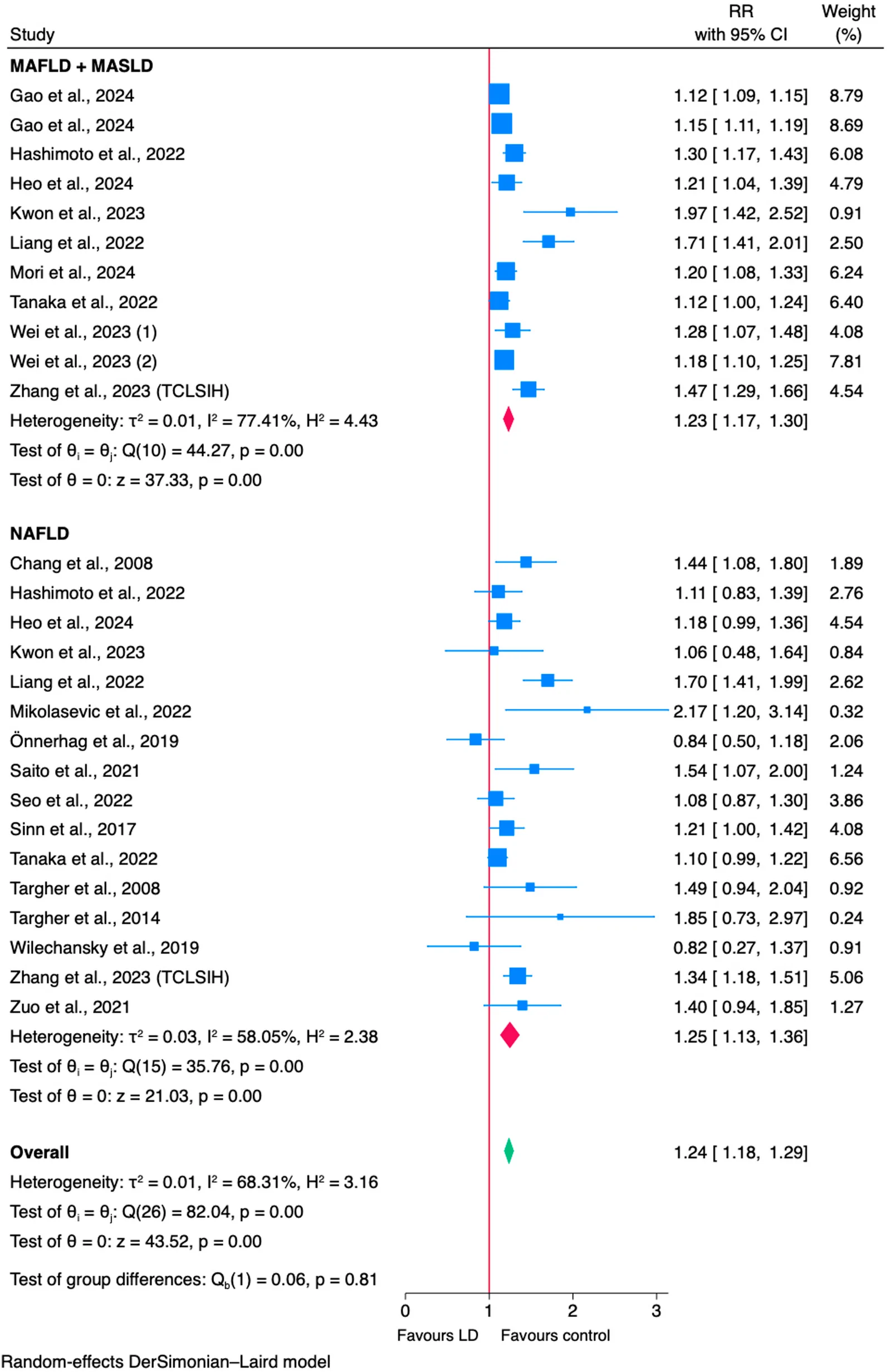
Forest plot comparing associations between NAFLD with CKD [22– [36, 38] *versus* MAFLD and MASLD with CKD [22, 26, 27, 31, 34], 37– [41]. CKD: Chronic Kidney Disease; NAFLD: Non-alcoholic Fatty Liver Disease; MAFLD: Metabolic Dysfunction-Associated Fatty Liver Disease; MASLD: Metabolic Dysfunction-Associated Steatotic Liver Disease

Considering the conceptual difference between the classification methods for liver disease of non-alcoholic and metabolic etiology, we performed a meta-analysis comparing the association of incident CKD between the group with NAFLD *versus* both groups, MAFLD and MASLD. The pooled RR of CKD in the group of NAFLD patients was 1.25 (95% CI 1.13–1.36), while in the MAFLD + MASLD group was 1.23 (95% CI 1.17–1.30) (Fig. 3).

### Sensitivity analyses and Quality assessment of selected studies

**Fig. 4:**
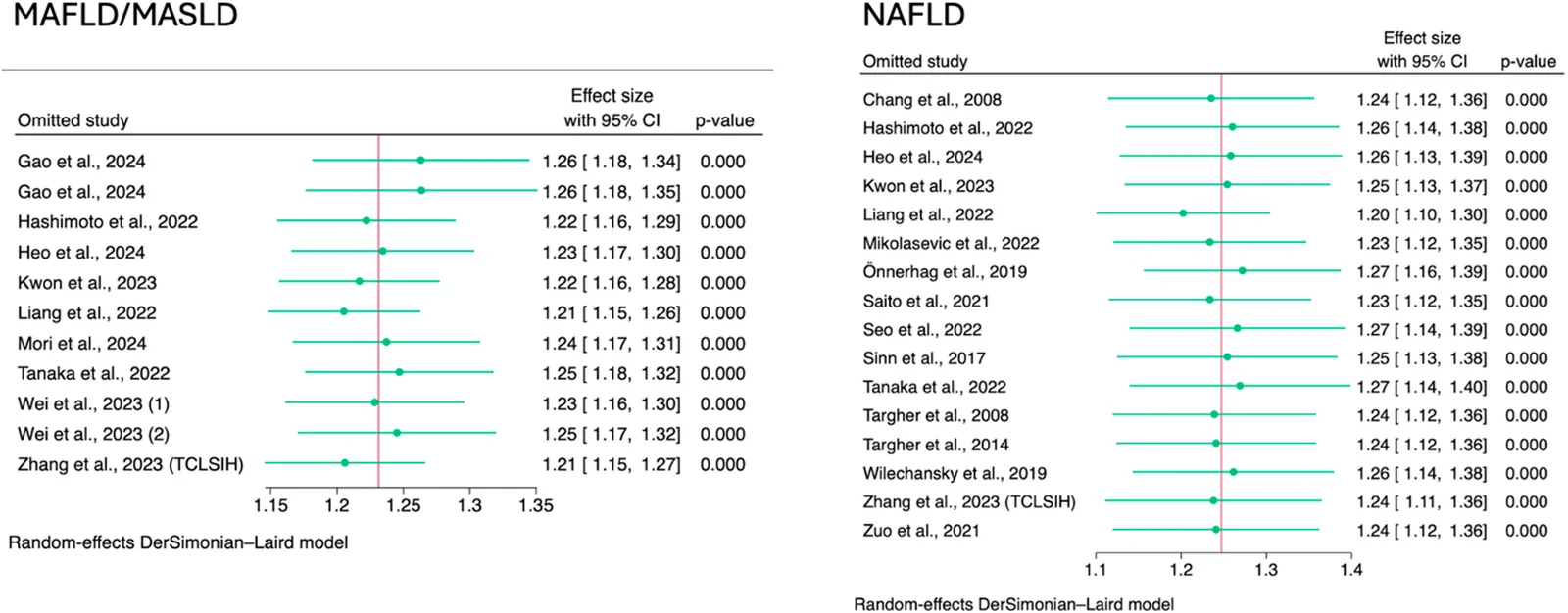
Sensitivity analysis

The changes observed in the sensitivity analyses were not significant, indicating that omitting studies did not alter the effect estimates for either exposure factor (Fig. 4). An analysis grouping studies addressing diabetics and studies with the general population was performed, and the result did not differ from previous analyses (Figure S1). Furthermore, an analysis was performed by grouping studies by the effect measure presented (OR, HR, or RR) for NAFLD and MAFLD/MASLD, and the results remained consistent across all scenarios (Figures S2 and S3).

**Fig. 5:**
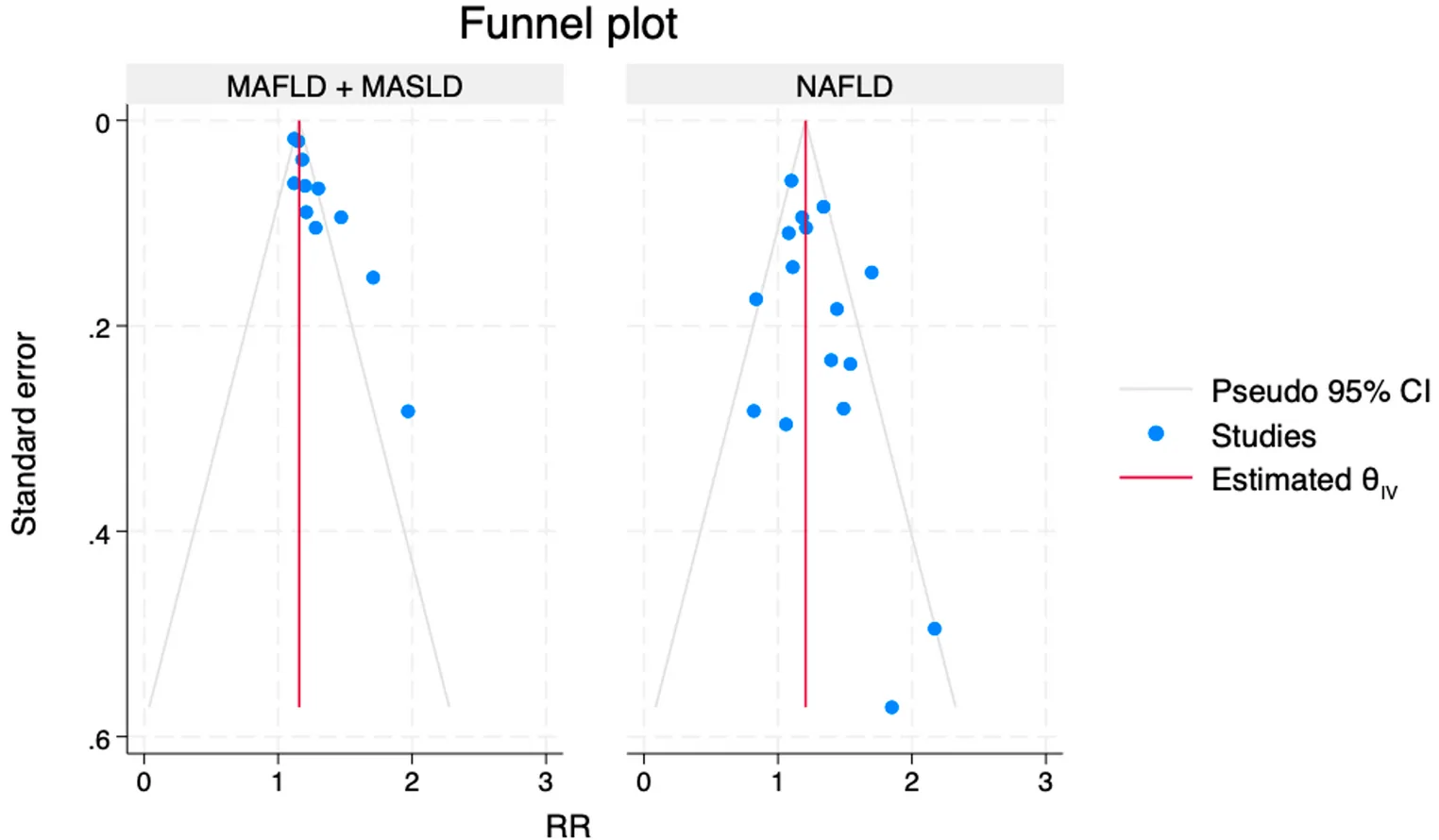
Funnel plot for the association of “MAFLD + MASLD” with CKD, and “NAFLD only” with CKD. CKD: Chronic Kidney Disease; NAFLD: Non-alcoholic Fatty Liver Disease; MAFLD: Metabolic Dysfunction-Associated Fatty Liver Disease; MASLD: Metabolic Dysfunction-Associated Steatotic Liver Disease

All articles included were assessed and deemed high quality, earning ratings between 8 and 9 stars (Table 2). Egger’s test indicated no small-study effects for NAFLD analysis. Also, Egger’s test was performed for analyses with fewer than 10 studies, and the results suggested no small-study effects (*p* > 0.05) for MAFLD and MASLD analyses. A funnel plot was performed by grouping the NAFLD and MAFLD/MASLD studies, and the results suggested no publication bias (Fig. 5).

**Table 2:** Quality assessment of studies included for meta-analysis

Quality assessment of studies (cohort)										
	Selection				Comparability		Outcome			Total Stars
Studies	1	2	3	4	1 A	1B	1	2	3	
Chang et al.	Yes	Yes	Yes	Yes	Yes	Yes	Yes	Yes	Yes	9
Gao et al.	Yes	Yes	Yes	Yes	Yes	Yes	Yes	Yes	Yes	9
Hashimoto et al.	Yes	Yes	Yes	Yes	Yes	Yes	Yes	Yes	Yes	9
Heo et al.	Yes	Yes	Yes	Yes	Yes	Yes	Yes	Yes	Yes	9
Kwon et al.	Yes	Yes	Yes	Yes	Yes	Yes	Yes	Yes	Yes	9
Liang et al.	Yes	Yes	Yes	No	Yes	Yes	Yes	Yes	Yes	8
Mikolasevic et al.	Yes	Yes	Yes	Yes	Yes	Yes	Yes	Yes	Yes	9
Mori et al.	Yes	Yes	Yes	Yes	Yes	Yes	Yes	Yes	Yes	9
Önnerhag et al.	Yes	No	Yes	Yes	Yes	Yes	Yes	Yes	Yes	8
Saito et al.	Yes	Yes	Yes	Yes	Yes	Yes	Yes	Yes	Yes	9
Seo et al.	Yes	Yes	Yes	Yes	Yes	Yes	Yes	Yes	Yes	9
Sinn et al.	Yes	Yes	Yes	Yes	Yes	Yes	Yes	Yes	Yes	9
Tanaka et al.	Yes	Yes	Yes	Yes	Yes	Yes	Yes	Yes	Yes	9
Targher et al. (2008)	Yes	Yes	Yes	Yes	Yes	Yes	Yes	Yes	Yes	9
Targher et al. (2014)	Yes	Yes	Yes	Yes	Yes	Yes	Yes	Yes	Yes	9
Wei et al. (2023)	Yes	Yes	Yes	Yes	Yes	Yes	Yes	Yes	Yes	9
Wei et al. (2023 – New)	Yes	Yes	Yes	Yes	Yes	Yes	Yes	Yes	Yes	9
Wilechansky et al.	Yes	Yes	Yes	Yes	Yes	Yes	Yes	Yes	Yes	9
Zhang et al.	Yes	Yes	Yes	Yes	Yes	Yes	Yes	Yes	Yes	9
Zuo et al.	Yes	Yes	Yes	Yes	Yes	Yes	Yes	Yes	No	8

## Discussion

This large meta-analysis found that MASLD and MAFLD were associated with an increased risk of incident CKD. We also performed a meta-analysis of studies using the nomenclature NAFLD, which lasted about 40 years until it was replaced by MAFLD in 2020, and obtained similar results.

Our findings indicate that the risk of developing CKD related to NAFLD and MAFLD/MASLD is similar in magnitude and statistically significant in both cases. It has been demonstrated that approximately 99% of patients with NAFLD meet the diagnostic criteria for MASLD criteria, which may reflect an overlap in the underlying pathophysiology of these entities. Accordingly, this suggests that the cardiometabolic risk factors incorporated into the MAFLD/MASLD definitions are already high prevalent among patients with NAFLD [42, 43].

The association between metabolic components of MAFLD/MASLD and CKD has been increasingly explored in recent years, highlighting potential injurious pathways linking these conditions. Insulin resistance, for instance, is considered a key contributor to microvascular remodelling that leads to tubular atrophy and interstitial fibrosis. Furthermore, obesity influences renal hemodynamics and structure by decreasing podocyte density and increasing foot process width, thereby contributing to the development of obesity-related glomerulopathy [44, 45]. 

It should be noted that the association is not straightforward to analyze, as these patients present one or more components of metabolic syndrome, which, in themselves, are common risk factors for CKD [46]. In fact, MASLD is a multisystemic disease whose pathogenesis arises from a complex interplay of genetic predisposition, environmental factors, and lifestyle determinants that interact with each other and with inflammatory pathways, increased oxidative stress, intestinal dysbiosis, liver and systemic toxicity, among other pathways [47–49]. It should be emphasized that the liver in MASLD is not merely a passive observer or simply a victim of accumulation within its structure. Instead, it actively contributes to the development and intensification of both hepatocellular and systemic damage observed in this condition [50–52].

In a very simplified way, although diet and lipogenesis contribute to the accumulation of free fatty acids (FFAs) in the plasma of patients with MASLD, the vast majority of these FFAs come from triglycerides present in visceral fat (catecholamines, via β-adrenergic pathway, and insulin resistance are the main drivers involved in this metabolic process). Due to the increase in plasma FFAs, there is upregulation of their membrane transporters, and they are internalized into hepatocytes. To address this excess of FFAs, the liver increases mitochondrial oxidation, thereby enhancing energy production, but also increasing oxidative stress. FFAs are also esterified to triglycerides, which are then stored as lipid droplets (steatosis) or, in a much smaller quantity, they are fused with apolipoprotein B-100 molecules to form a VLDL particle, which is exported from the liver. FFAs also generate lipotoxic products (ceramides, diacylglycerol, acylcarnitines), leading to insulin signaling dysfunction and hepatocellular damage [47, 51–54].

Regarding the association of an increased incidence of CKD in MASLD patients, it may be at least in part explained by factors directly produced by the liver that may influence patients’ metabolic syndrome components. Increased liver production of inflammatory cytokines (TNF-α, IL-1β, IL-6) and hepatokines (such as fibroblast growth factor 21- FGF21-, angiopoietin-like proteins and fetuins-A&B) augment insulin resistance, inflammation, and oxidative stress. Furthermore, direct lipotoxicity to renal tubules and cells, impaired antioxidant defense and consequent oxidative stress [55–60]; dysbiosis [56, 58, 59, 61, 62]; and PNPLA3, TM6SF2, MBOAT7 and GCKR genotypes are also mechanisms considered by some reviews as potentials links between MASLD and CKD [56, 62].

In 2014, the first meta-analysis concerning this association was published by Musso et al., stating that NAFLD should be a target for the prevention and treatment of CKD after finding a positive association between the presence and severity of NAFLD and the increased risk and severity of CKD (HR 1.79, 95% CI 1.65–1.95) [57]. Thus, while our meta-analysis included exclusively 20 longitudinal cohorts with the primary outcome being incident CKD and had a total sample of 498,955 individuals, this previous one included twenty-three studies, of which only 13 were longitudinal studies targeting incident CKD, consequently totaling a sample of 28,963 individuals.

In 2020, Mantovani et al. demonstrated a significant association between SLD and long-term risk of incident CKD stage ≥ 3 with a HR 1.43 [95% CI 1.33–1.54]. However, the majority of the studies included used neither imaging nor biopsy to assess liver steatosis [63].

A previous meta-analysis by Mantovani et al. included only longitudinal studies with incident CKD as the outcome, comprising 9 observational studies that diagnosed SLD based on liver enzymes, and/or US, and/or fatty liver index (FLI). In total, 96,595 adult individuals were analyzed (34.1% with NAFLD; *n* = 32,898), with 4653 cases of incident CKD [64]. In comparison, despite applying more rigid inclusion criteria - prioritizing histopathological and imaging assessment of SLD - our study included a substantially larger sample of approximately 498,955 individuals, with 66,309 incident CKD cases. The decision to prioritize US was based on its wide availability and diagnostic accuracy to detect hepatic steatosis. Hernaez et al. demonstrated that US achieves a sensitivity of 93.6% and a specificity of 80.1% for detecting ≥20–30% steatosis when compared with other imaging modalities such as CT and MRI [65].

Moreover, a recent cohort study published by Heo et al. diverges from previous data. The authors found no increased risk for either incident CKD or development of abnormal albuminuria in patients with NAFLD. However, patients with a diagnosis of MASLD, the last proposed nomenclature in 2023, did have a significantly increased incidence of CKD, mainly due to the high prevalence of abnormal albuminuria [22]. 

The recognition that the incidence of MASLD increases year by year and the understanding of its clinical consequences are of fundamental importance for physicians and other health professionals, as well as for family members and the general public, since basic health measures, such as combating obesity and a sedentary lifestyle, are very useful in preventing or minimizing the effects of this condition. In addition to lifestyle changes, new obesity drugs such as the Glucagon-Like Peptide-1 (GLP-1) receptor agonists and the dual Gastric Inhibitory Peptide (GIP)/GLP-1 receptor co-agonist (Tirzepatide), offer a positive perspective for the management of MASLD, mainly by promoting significant weight loss amongst other metabolic effects [66]. In fact, very recently, a GLP-1 agonist (Semaglutide, Wegovy) received FDA approval for the treatment of metabolic dysfunction–associated steatohepatitis (MASH), which represents the progressive form of MASLD [67]. Previously, the FDA had already given approval to Resmetirom, an oral liver-directed, thyroid hormone receptor beta–selective agonist, that act by reducing intrahepatic triglycerides content, for the same purpose [68, 69].

This study has several strengths. To our knowledge, this is the meta-analysis of longitudinal studies involving the largest number of patients. This study may help general practitioners, hepatologists, nephrologists, and several other medical and healthcare professionals to be more alert to the association and to identify and treat patients affected by both conditions even more effectively, as an interdisciplinary approach to these patients will probably be required to achieve the best possible results.

The limitations of this study include the rigid barrier created by our inclusion criteria, which not only selected studies with incident CKD as the outcome but also excluded many studies that used non-invasive methods to assess SLD, such as FLI. Additionally, substantial heterogeneity was observed across studies, mainly in MAFLD, which may be explained by differences in study populations, follow-up durations, and variability in diagnostic methods for steatotic liver disease. The vast majority of included studies relied on conventional US rather than more advanced techniques, such as vibration-controlled transient elastography. Finally, although we assessed included studies for potential duplication, a degree of population overlaps, particularly among studies conducted by the same authors, cannot be entirely excluded.

## Conclusion

This large meta-analysis demonstrated that the presence of SLD is associated with an increased risk of CKD, regardless of whether the older terminology (NAFLD) or the more recent one (MASLD) is used. This finding was expected, given the substantial overlap in metabolic factors—estimated at 90–95%—between the two classifications. However, the term MASLD emphasizes the metabolic components underlying the condition and is currently the preferred nomenclature.

It is important to highlight that NAFLD/MASLD represents a preventable and treatable risk factor for CKD. Given the continuous rise in the prevalence of both NAFLD/MASLD and CKD, awareness of this association is crucial, as it may contribute to the effective management of a large number of patients. Further studies are needed to better understand the role of individual metabolic risk factors, as well as specific combinations of these factors, in the development of CKD.

## Supplementary Information

Below is the link to the electronic supplementary material.

Supplementary Material 1 (DOCX 662 KB)

## Data Availability

No datasets were generated or analysed during the current study.
