# Supplementary material for: Association between metabolic dysfunction-associated steatotic liver disease/non-alcoholic fatty liver disease and chronic kidney disease: a systematic review and meta-analysis
Source: Rev Endocr Metab Disord. 2026 Jun 11;27(5):999–1021. doi: 10.1007/s11154-026-10059-0 (PMC13616005; doi:10.1007/s11154-026-10059-0)
Supplement: Supplementary file 1 [file 11154_2026_10059_MOESM1_ESM.docx]

**Supplementary material**

**Search estrategieis**

For PubMed and Cochrane, Boolean search strategies were applied. However, for the LILACS and SciELO databases, studies were identified through manual searches on the BVS platform, combining terms individually using the AND operator and applying filters specific to LILACS and SciELO.

1. **PubMed: 1,715 results**

| **Line** | **Search strategy** |
| --- | --- |
| **#1** | (Non-alcoholic fatty liver disease OR Nonalcoholic Fatty Liver OR NAFLD OR non-alcoholic steatohepatitis OR MASLD OR metabolic dysfunction-associated steatotic liver disease) |
| **#2** | (Renal insufficiency, chronic OR chronic kidney disease OR Kidney diseases OR Kidney failure OR estimated glomerular filtration rate) |
| **#3** | #1 AND #2 |

2. **Lilacs/SciELO: 179 results**

| **Line** | **Search Strategy** |
| --- | --- |
| **#1** | (Non-alcoholic fatty liver disease) |
| **#2** | (Nonalcoholic Fatty Live) |
| **#3** | (NAFL) |
| **#4** | (non-alcoholic steatohepatitis) |
| **#5** | (MASL) |
| **#6** | (metabolic dysfunction-associated steatotic liver disease) |
| **#7** | (Renal insufficiency) |
| **#8** | (chronic kidney disease) |
| **#9** | (Kidney diseases) |
| **#10** | (Kidney failure) |
| **#11** | (estimated glomerular filtration rate) |
| **#12** | #1 AND #7, #1 AND #8, #1 AND #9, #1 AND #10, #1 AND #11 |
| **#13** | #2 AND #7, #2 AND #8, #2 AND #9, #2 AND #10, #2 AND #11 |
| **#14** | #3 AND #7, #3 AND #8, #3 AND #9, #3 AND #10, #3 AND #11 |
| **#15** | #4 AND #7, #4 AND #8, #4 AND #9, #4 AND #10, #4 AND #11 |
| **#16** | #5 AND #7, #5 AND #8, #5 AND #9, #5 AND #10, #5 AND #11 |
| **#17** | #6 AND #7, #6 AND #8, #6 AND #9, #6 AND #10, #6 AND #11 |

3. **Cochrane: 205**

| **Line** | **Search Strategy** |
| --- | --- |
| **#1** | (Non-alcoholic fatty liver disease OR Nonalcoholic Fatty Liver OR NAFLD OR non-alcoholic steatohepatitis OR MASLD OR metabolic dysfunction-associated steatotic liver disease) |
| **#2** | (Renal insufficiency, chronic OR chronic kidney disease OR Kidney diseases OR Kidney failure OR estimated glomerular filtration rate) |
| **#3** | #1 AND #2 |

**Subgroups Analysis**

Figure S1. Risk of CKD incidence among LD patients by type of population.

Figure S1. Risk of CKD incidence among MAFLD/MASLD patients by effect measure.

Figure S2. Risk of CKD incidence among NAFLD patients by effect measure.
